# Rethinking Administrative Law for Algorithmic Decision Making

**DOI:** 10.1093/ojls/gqab032

**Published:** 2021-10-30

**Authors:** Rebecca Williams

**Keywords:** algorithmic decision making, judicial review, artificial intelligence, machine learning

## Abstract

The increasing prevalence of algorithmic decision making (ADM) by public authorities raises a number of challenges for administrative law in the form of technical decisions about the necessary metrics for evaluating such systems, their opacity, the scalability of errors, their use of correlation as opposed to causation and so on. If administrative law is to provide the necessary guidance to enable optimal use of such systems, there are a number of ways in which it will need to become more nuanced and advanced. However, if it is able to rise to this challenge, administrative law has the potential not only to do useful work itself in controlling ADM, but also to support the work of the Information Commissioner’s Office and provide guidance on the interpretation of concepts such as ‘meaningful information’ and ‘proportionality’ within the General Data Protection Regulation.

## 1. Introduction

The controversy over the algorithm used to generate A level grades^1^ has again brought to the forefront of attention the increasing role played by algorithms in public decision making,^2^ and from Cathy O’Neill’s description of ‘Weapons of Math Destruction’^3^ (WMDs) to Frank Pasquale’s concerns about ‘black boxes’,^4^ we are well aware of the dangers they can pose. While there is evidence, both in the case of A levels and in other contexts,^5^ that public authorities may be limiting their use of such systems, at the same time, with increasing pressure on public finances and the ability of such systems to make decisions more cheaply than humans, it seems unlikely even after the A level debacle that they will disappear from use altogether.^6^ Nor would we necessarily want them to; while the A level algorithm was a problematic response to a problem raised by the pandemic,^7^ in contexts such as medicine machine learning has been at the forefront of research and development.^8^ The purpose of this article is therefore to consider how public law in particular can respond to the challenges of automated decision making (ADM) by public authorities in a way that will optimise their use, enabling us to harness their advantages while avoiding the risks to which they may give rise.^9^

## 2. What Is an Algorithm?

Before we look at public law specifically, it is worth recalling that algorithmic decision making is not new. Hill describes algorithms as ‘mathematical constructs’ with ‘a finite, abstract, effective, compound control structure, imperatively given, accomplishing a given purpose under given provisions’.^10^ The Law Society’s review of algorithms in the criminal justice system cites the ‘classic’ computer science definition that it is ‘any well-defined computational procedure that takes some value, or set of values, as input and produces some value, or set of values, as output’.^11^ Thus, even a simple decision tree drawn by hand on a piece of paper can constitute algorithmic decision making,^12^ and such algorithms have often been used by public authorities.^13^ This latter kind of algorithm is used in so-called artificial intelligence (AI) ‘expert systems’, which are also, unsurprisingly, known as ‘rules-based systems’. These systems are complex but transparent, and those programming them have complete control over the precise set of ‘if–then’ rules they follow.

However, a subset of AI known as machine learning (ML) uses an algorithm to build a mathematical model from the data upwards, rather than constructing all the relevant rules from the top down, as rules-based systems do. There are a variety of forms of ML, including ‘deep learning’, which derives its name from the fact that the algorithm’s structure comprises multiple connected layers. An input layer contains the initial data, an output layer produces the result for the given inputs and in between there are a series of hidden layers made up of interconnected ‘nodes’. The interconnection of these nodes is what gives this form of deep learning the name ‘artificial neural network’, as a reference to the (in fact quite different) idea of a biological neural network.^14^ These forms of algorithm are far less transparent than rules-based or expert systems,^15^ but there are ways in which their performance can be assessed.

## 3. Assessing ML Performance

**Figure 1:**
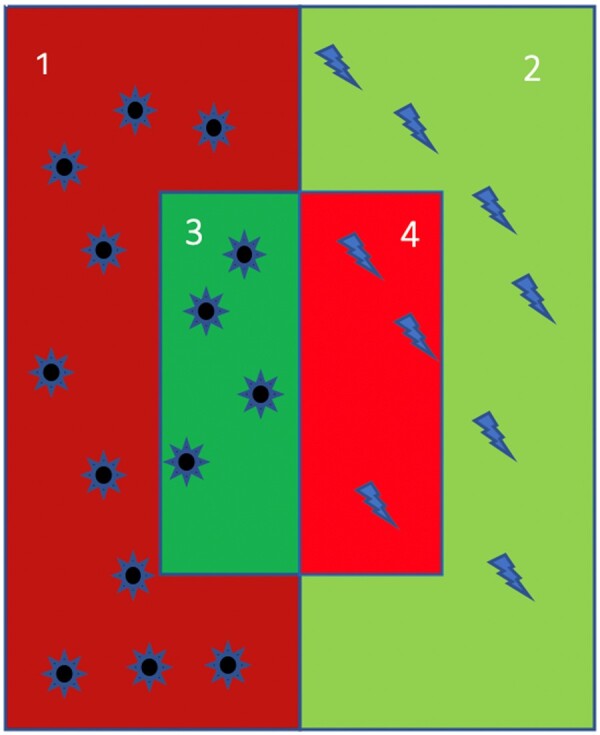
Illustratio n of example results from a shape identification system.

Imagine a system has learned (whether or not in a supervised way) to detect sun shapes, while rejecting lightning bolts. The system is then given a series of shapes to identify and the outcome is presented in Figure  1.

All the shapes in areas 1, 2, 3 and 4 are shown to the system. The system identifies as suns the items in the smaller rectangle (3 + 4). Area 1 therefore contains suns which are missed by the system (false negatives). Area 2 contains lightning bolts which are correctly rejected by the system (true negatives). Area 3 contains suns which are correctly recognised by the system (true positives) and area 4 contains lightning bolts which are incorrectly identified as suns by the system (false positives). There are various ways in which the accuracy of this system could be measured.

For example, the ‘sensitivity’ (or ‘true positive rate’)^16^ of the system is a measure of how many of the target items were identified. So the sensitivity for this system would be 4/14; the system has correctly identified 4 suns (in area 3) out of a total of 14 possible suns (areas 1 + 3). This can also be thought of as the probability of detection.

Conversely, the ‘specificity’ of a system is also known as the ‘true negative rate’, identifying how many items were correctly rejected. So the ‘specificity’ of this system would be 2/3 because it has correctly rejected 6 (in area 2) out of 9 lightning bolts (in areas 2 + 4).

A third metric, known as ‘precision’, assesses how many of the positive responses were true, as opposed to false positives. So for this system the precision would be 4/7. The system has identified 7 items (areas 3 + 4), but only 4 of them are true positive suns.

A fourth metric, known as the ‘negative predictive value’, assesses how many of the negative responses were true, as opposed to false negatives. So for this system it would be 6/16.

‘Accuracy’, on the other hand, measures the overall performance of the system. So in this case it would be the number of correctly classified items in areas 3 + 2 (10) as a proportion of all the items in all the areas (23), i.e. 10/23. It might be thought that accuracy would always be the best metric for assessing a particular system, but of course this will depend on which errors are the most serious. If the model above were really about detecting cancer in patients, then the number of false negatives (ie missed suns in area 1) might be more problematic than the incorrectly identified lightning bolts in area 4, and thus the sensitivity of the system might be more significant than its overall accuracy.

## 4. Is Algorithmic Decision Making Problematic?

It does not follow that algorithmic decision making is problematic. Indeed, as noted above, sometimes it is quite the opposite, for example if an algorithm can be taught to identify cancerous as opposed to non-cancerous cells more rapidly than can a human being.^17^ Even O’Neill, who states that she is not a ‘big data evangelist’^18^ and devotes her book to ‘focus[ing] sharply in the other direction, on the damage inflicted by WMDs and the injustice they perpetuate’,^19^ suggests that she would use data to discover the impact of solitary confinement on recidivism,^20^ and notes the positive impact data-driven decision making can have on baseball selections.^21^ And it is not as if human beings are flawlessly accurate either. They can base their decisions on hunches,^22^ have conscious or unconscious prejudices, be affected by their own human experiences and emotions, such as the performance of their favourite football team,^23^ or act inconsistently.^24^ In short, it is hard to argue that better-informed and more systematic, consistent and coherent decision making is a step in the wrong direction.^25^ Nonetheless, there are various ways in which public law will need to adapt in order to provide optimal control of such systems.

## 5. Legislation

### A. Permitting ADM in the First Place

The first issue is whether it is even possible for a public authority to use ADM. Article 22 of the General Data Protection Regulation^26^ states that data subjects (DSs) have the right not to be subject to a decision based solely on automated processing, including profiling, which produces legal effects concerning them or similarly significantly affects them, unless it has a legal basis. In general, such a legal basis can be provided by consent (article 22(2)(c)), but it seems unlikely that this will be an option for public authorities since Recital 43 to the GDPR states that

consent should not provide a valid legal ground for the processing of personal data in a specific case where there is a clear imbalance between the data subject and the controller, in particular where the controller is a public authority and it is therefore unlikely that consent was freely given in all the circumstances of that specific situation.

Recitals are, of course, not legally binding, but nor are they completely irrelevant, the ECJ having stated that ‘whilst a recital in the preamble to a regulation may cast light on the interpretation to be given to a legal rule, it cannot in itself constitute such a rule’.^27^ It therefore seems advisable for public authorities to err on the side of assuming that ADM will require Member State or EU authorisation in legislation ‘which also lays down suitable measures to safeguard the data subject‘s rights, freedoms and legitimate interests’ (article 22(2)(b)). Where the processing in question deals with article 9 sensitive data (revealing racial or ethnic origin, political opinions, religious or philosophical beliefs and trade union membership, or genetic, biometric, health or sexual data), processing must be necessary for reasons of substantial public interest, be on the basis of law, be proportionate to the aim pursued, respect the essence of the right to data protection and again provide suitable and specific measures to safeguard the fundamental rights and interests of the DS (article 22(4) GDPR).

From January 2021 the GDPR has been contained in the UK GDPR.^28^ In any event, in existing domestic law, section 14 of the Data Protection Act (DPA) 2018 incorporates article 22(2)(b),^29^ while section 49 provides that a law enforcement controller may not take a significant decision based solely on automated processing unless that decision is required or authorised by law, and that a decision is a ‘significant decision’ if it either produces an adverse legal effect concerning the DS or significantly affects them. A similar provision is made for intelligence purposes in section 96.

The conclusion, therefore, is that if public authorities wish to rely wholly on ADM they will require some kind of specific legal basis to do so, which must lay down protections for the DS.

### B. Rights Applicable under ADM

If the public authority does use ADM, this gives rise to further rights on the part of the DS. First, at the time the data are obtained from the DS, article 13(2)(f) GDPR gives the DS a right to know the existence of the ADM, including profiling; a right to ‘meaningful information about the logic involved’; and the right to know the significance and envisaged consequences of such processing for the DS. Recital 71 adds that this should include ‘an explanation of the decision reached after such assessment’. These are passive rights in that the data controller must give them this information spontaneously. If the data later change hands and are again to be used for ADM, the DS has the right to the same information, now via article 14(2)(g), either within one month of the data’s receipt by the new controller (article 14(3)(a)) or at the time of first communication with the DS (article 14(3)(b)), whichever is the sooner. This is also a passive right which the data controller must fulfil spontaneously. In addition to these rights, at any point, article 15(1)(h) also gives the DS the right to obtain this information actively. As the Article 29 Working Party (now known as the European Data Protection Board (EDPB)) points out, this is really a ‘belt and braces’ provision because the DS ought already to have been given that information via the passive rights of article 13 or 14.^30^ Article 22 also gives individuals ‘at least the right to obtain human intervention on the part of the controller’ to express their point of view and to contest the decision.

The question then is what precisely is meant by ‘meaningful information about the logic involved’ and the academic debate on this point is well known. Goodman and Flaxman had initially argued that this provision conferred a right to a ‘full explanation’, which would limit the ability to use ML that acts in a more opaque manner.^31^ This argument was then countered by Wachter, Mittelstadt and Floridi, who argued that ‘the right of access … only grants access to an *ex ante* explanation of system functionality… [I]t is reasonable to doubt that the right of access grants a right to *ex post* explanations of *specific decisions* already reached’.^32^ A third view, expressed by Selbst and Powles, argued instead that the right should be interpreted functionally and flexibly, and at a minimum should enable DSs to exercise their GDPR and human rights.^33^ A similar view is given by Kaminski,^34^ and this perspective also chimes with the conclusion of the EDPB that ‘the information provided should … be sufficiently comprehensive for the data subject to understand the reasons for the decision’, such as ‘details of the main characteristics considered in reaching the decision, the source of this information and the relevance’.^35^ The flexibility and context specificity of the explanation is emphasised also by the Information Commissioner’s Office (ICO) and Alan Turing Institute’s report,^36^ which identifies six types of explanation: the rationale (reasons leading to a decision); responsibility (who is involved and whom to contact); an explanation of which data have been used and how; an explanation of how equal and equitable treatment is to be ensured; an explanation of the system’s safety and performance in terms of reliability, security and robustness; and finally an explanation of the potential impact of the system on individuals and society.^37^ They explain:

Our own research reveals that context is a key aspect of explaining decisions involving AI … Therefore when we talk about explanations … we do not refer to just one approach … or to providing a single type of information… Instead, the context affects which type of explanation you use to make an AI-assisted decision clear or easy for individuals to understand.^38^

Whereas to trigger article 22 the decision had to be *solely* based on automated processing, as the EDPB notes, article 4(4) GDPR defines profiling as

any form of automated processing of personal data consisting of the use of personal data to evaluate certain personal aspects relating to a natural person, in particular to analyse or predict aspects concerning that natural person’s performance at work, economic situation, health, personal preferences, interests, reliability, behaviour, location or movements.

Thus, for the EDPB, as long as profiling involves some form of automated processing, human involvement does not necessarily take the activity out of the definition. The rights under articles 13–15 are therefore triggered as long as there is some form of automated processing in the decision, whether or not the decision is based solely on that automated processing (triggering article 22) or not.^39^ In the case of solely automated processing based on consent or contract, article 22 gives individuals ‘at least the right to obtain human intervention on the part of the controller to express his or her point of view and to contest the decision.’ This does not specifically apply to law-based processing, which, as noted above, is likely to be the applicable justification for public authorities. However, it seems more likely that this is because it is anticipated that such protections will be laid down in the ‘suitable measures to safeguard the data subject’s rights, freedoms and legitimate interests’ (article 22(2)(b)), which must also be contained in such a law, rather than because the GDPR anticipates the absence of such protections altogether. This is confirmed by the EDPB, which states that ‘such measures should include as a minimum a way for the data subject to obtain human intervention, express their point of view and contest the decision’.^40^

Domestic legislation largely mirrors this by providing in DPA 2018, section 14 that where a significant decision based solely on automated processing is required or authorised by law, the controller must as soon as reasonably practicable notify the DS in writing that such a decision has been taken. The DS then has one month to request the controller either to reconsider the decision or to take a new one not based solely on automated processing, thus triggering a further month for the controller to consider, comply and inform the DS. The Secretary of State can also make further regulations to provide additional measures to safeguard the DS’s rights, freedoms and legitimate interests. Similar provisions for law enforcement and intelligence are contained in sections 50 and 97 respectively.

### C. The Public Sector Equality Duty and the Human Rights Act 1998

In addition, the Equality Act 2010, section 149(1) states that a public authority in the exercise of its functions must have due regard to the need to eliminate discrimination, harassment, victimisation and any other conduct prohibited by the 2010 Act, and must both advance equality of opportunity and foster good relations between those who share a relevant protected characteristic and those who do not. Section 1(3) further states that having due regard to the need to advance equality of opportunity involves having due regard in particular to the need to remove or minimise disadvantages suffered by persons who share a relevant protected characteristic that are connected to that characteristic, to take steps to meet the different needs of such persons and to encourage such persons to participate in public life or other activity in which participation by such persons is disproportionately low. The relevant protected characteristics are age, disability, gender reassignment, pregnancy and maternity, race, religion or belief, sex and sexual orientation. Protection is, of course, also provided by the Human Rights Act 1998.

Both these forms of protection played a significant role in the decision in *Bridges*,^41^ one of the first instances of judicial review to tackle some of these issues.^42^ In this case, Mr Bridges argued that the use of automated facial recognition (AFR) technology by South Wales Police (SWP) was unlawful. The Court of Appeal agreed, holding that the use of the technology was not ‘in accordance with the law’ for the purposes of article 8(2) of the European Convention on Human Rights (ECHR)^43^ because there was no clear guidance on where the technology could be used and who could be put on a watchlist. The Court also found that the force had not done all that it reasonably could to discharge the duty placed on it by the Public Sector Equality Duty (PSED).

At first instance in *Bridges* the court had heard the evidence of a police officer relating to AFR Locate deployments from after the UEFA Champions League Final of 2017 through to June 2018. During those deployments, 290 alerts were generated. Eighty-two were true positives and 208 were false positives.^44^ These statistics applied only to those who generated an alert. The identity of those who passed the camera without generating an alert was not known. The court went on to hear that

[188] 188 of the alerts were males (65%). Of the 188 male alerts, 64 (34%) were true positives and 124 (66%) were false positives. In relation to females, of 102 alerts, 18 (18%) were true positives and 84 (82%) were false positives. A number of the female false alerts were matched against primarily two individuals who the AFR software provider would refer to as a ‘lamb’. A lamb is a person whose face has such generic features that [it] may match much more frequently.

The same police officer also reviewed the ethnicity of those who were the subject of an alert. Of the true positives (82), 98% were ‘white north European’. Of the false positives (208), 98.5% were ‘white north European’. The officer therefore concluded that from his experience and the information available to him he had seen no bias based on either gender or ethnicity.

However, the first instance court had also heard the expert evidence of Dr Jain, a US professor of computer science, to the effect that a key factor in the accuracy of such a system would be the *accuracy of its training data* (by which he presumably meant how representative the training data are of the true distribution of examples in the world or whether they are otherwise skewed in some way). It appeared that SWP were not aware of the data used to train the system, meaning that they had no means of establishing whether or not there was bias. Conversely, the evidence of a representative of the company providing the AFR technology used by SWP was that the system had been trained on data containing roughly equal numbers of male and female faces and a wide range of ethnicities, although for reasons of commercial sensitivity more detail about the data was not given. Dr Jain’s response to this was that without the ability to do a thorough evaluation of the demographic composition of the algorithm’s training dataset SWP would not be able to assess whether the training dataset was or might be biased.

On appeal, the Court of Appeal made two points in relation to this evidence. One was that the officer in question was not a technical expert in the relevant software, by which they presumably meant in data science. And although the Court of Appeal held that ‘it is not the role of this Court to adjudicate on the different points of view expressed by [the two witnesses]. That would not be appropriate in a claim for judicial review, still less on appeal’,^45^ it nevertheless went on to do something very similar. And indeed it is difficult to see how this could have been avoided. Simply stating that the SWP had not gone far enough, without any indication of what further actions might have been needed, would not have been helpful. Yet, in order to provide that guidance, it was inevitable that the Court in *Bridges*, as will no doubt be the case with courts in future judicial reviews of this kind, had to engage at a detailed level with the technical evidence.

On the substance, in providing that guidance, the second point made by the Court of Appeal was that, while it understood that the immediate deletion of the data of those who did not generate an alert was an integral data protection safeguard, that did not mean the software did not have an inbuilt bias that needed to be assessed and which could be assessed only by having access to these data. It went on to hold that SWP had not sought to satisfy themselves, either directly or by way of independent verification, that the software program in this case did not have an unacceptable bias on grounds of race or sex. While the Court understood the concerns about commercial confidentiality, the resulting lack of information (which presumably would also include the training dataset) ‘does not enable a public authority to discharge its own, non-delegable duty under section 149’.^46^ And indeed earlier the Court had concluded that ‘As a minimum for confirming whether an AFR system is biased, the database statistics, such as the number of males to females, and different races considered, would need to be known’.^47^

It might thus be thought that if a public authority wishes to use such an algorithm, it will in the first instance have to acquire these data as part of its contract with the private provider of the system. But since this may be commercially unrealistic, there appears to be a pressing need to provide an alternative method of complying with the PSED. As the evidence of Dr Jain suggests, access to the actual data is not necessary as long as there is ‘some kind of thorough evaluation of the demographic composition of the algorithm’s training dataset’. But the dangers here are that the public authority would have to trust the dataset’s owner to provide this information accurately, and conversely the dataset owner might be concerned that to give away too much of this information would come too close to revealing the actual data. It seems preferable, then, for there to be some form of regulatory verification, whereby the verifying entity could have access to the commercially sensitive data (as does the patent office, for example) in order to carry out the necessary checks. Use of a system which had received a ‘kitemark’ from such an entity would then comply with the PSED. At present, however, there is no such system in place, and indeed *Bridges* only deals with one set of tests that need be run.

## 6. The Common Law

As the section above shows, it has so far been statutes that have both provided for and been used to target public authority ADM, but it seems likely that in future the common law rules of judicial review will also play a role.^48^

It is well known that judicial review acts as a referee, intervening only when a public authority has breached a particular legal rule, not simply when the court disagrees with the outcome of the game.^49^ The rules in question are that the public authority must have jurisdiction to make the decision;^50^ it must not fetter or delegate its discretion;^51^ it must follow the right procedure, meaning that it must not be biased; it must hear from the right people and it might have a duty to give reasons for its decision;^52^ it must take into account all the right and only the right considerations in making its decision;^53^ and its final decision must be reasonable or proportionate.^54^ However, while these are all grounds of review that can be used *against* a public authority, judicial review need not be, and indeed should not be, wholly antithetical to the interests of public authorities. The aim of judicial review as a ‘judge over your shoulder’ is intended to provide positive *ex ante* guidance, as well as challenging conclusions *ex post*. Enhancing the lawfulness of decisions made by public authorities can have the result of increasing public confidence and trust in them, thereby making it easier for the public authority to obtain the cooperation and obedience of the public. As the ICO and Alan Turing Institute’s guidance explains, this is particularly so in the context of ADM, where ‘the more insight individuals have on the AI model that makes decisions about them, the more confident they are likely to be in interacting with these systems and trusting … use of them’.^55^

## 7. Linking the Two Sets of Rules

Not only might these common law rules be relevant in their own right, there are also two different ways in which they are linked to the legislation discussed above. First, administrative law has specifically developed in order to control decision-making processes, making it an ideal place to look for definitions of expressions such as ‘suitable measures to safeguard the data subject’s rights, freedoms and legitimate interests’ (article 22(2)(b) GDPR) and ‘meaningful information about the logic involved’ (article 13(2)(f) GDPR). Indeed, sometimes this role is effectively mandated by the legislation. For example, the GDPR frequently refers to the concept, very familiar to administrative law, of ‘proportionality’, suggesting among other things that processing of sensitive data for substantial public interest, archiving, scientific or historical research purposes must be ‘proportionate to the aim pursued’.^56^ Secondly, those concerned by a particular ADM decision will refer the issue initially to the ICO, and the decision then reached by the ICO can be challenged through the General Regulatory Chamber of the First-tier Tribunal and ultimately by judicial review.^57^

It seems highly likely, then, that the common law of judicial review will have an important role to play both through the relevant legislation and in addition to it, whether by informing review of and appeals from the ICO or through review of the public law use of ADM directly. The question then is how these grounds of review should be adjusted in order to ensure the optimal development of ADM.

## 8. Judicial Review and ADM

### A. Following the Right Procedure

Although non-legal discussions of ADM often refer to the idea of ‘bias’, it is in fact obvious to public lawyers that this ground in its technical sense in administrative law is unlikely to be helpful, given its very specific focus on vested interests.^58^ However, administrative law has often in the past sought to control not just the fairness of an individual system once that system has been chosen by the public authority, but in fact the very choice of the particular decision-making system in the first place.^59^ If this continues, it seems very likely that courts in the future will be called upon to review the choice of ADM, the extent of its deployment and, in particular, the choice of one particular form of ADM as opposed to another. The ICO has helpfully set out a series of different forms of ADM and the extent to which, for example, they are each explainable,^60^ adding the advice that ‘The model you choose should be at the right level of interpretability for your use case and for the impact it will have on the decision recipient’.^61^ It is therefore by no means unforeseeable that courts in the future will be asked to review such choices in order to establish whether the choice amounted to a fair procedure. On this front, it is noteworthy that the *Ivory Coast*^62^ case, discussed below, considered the doctrine of fairness as a countervailing consideration in establishing how rigidly the policy could be applied in that case. Here too, then, the courts will have to develop both the technical expertise to understand the differences between the different systems and a justifiable set of principles for deciding which system is most appropriate in which case. The history of administrative law in this context has tended to be, inevitably, that the more court-like a procedure looks, the more fair it is held to be (especially given the references in article 6 ECHR to ‘independent and impartial’ tribunals), but this approach is unlikely to be nuanced enough to do anything other than simply reject the application of ADM. If we are to harness the potential benefits of these systems but also ensure that they are chosen and operated fairly, public law will have to develop a more advanced means for matching appropriate systems to contexts.

One closely connected ground of review for procedural fairness which will enable the courts to do this is the law regarding transparency and reason giving. It seems very likely that this will inform what is meant by ‘meaningful information about the logic involved’ as courts come to interpret that provision, as well as potentially applying this ground of review on its own terms.

Two sets of rules deal with these issues: those regarding the duty to give reasons (if any) after the decision^63^ and those specifying the claimant’s right to have notice of the case against them for the purposes of a fair hearing.^64^ The two are often connected in the sense that reasons given after one decision are often instrumentally relevant to any subsequent appeal, at which point they become more akin to notice. This is also reinforced by the way in which they are treated by the ICO guidance, which states that:

It is vital that individuals understand the reasons underlying the outcome of an automated decision, or a human decision that has been assisted by the results of an AI system. If the decision was not what they wanted or expected, this allows them to assess whether they believe the reasoning of the decision is flawed. If they wish to challenge the decision, knowing the reasoning supports them to formulate a coherent argument for why they think this is the case.^65^

The duty to give notice in particular is well established at common law, and even in the context of closed material procedures (CMPs) it has been held that the defendant must often be told the ‘gist’ of the case against them.^66^ In *Bourgass*,^67^ Lord Reed held that:

a prisoner’s right to make representations is largely valueless unless he knows the substance of the case being advanced … That will not normally require the disclosure of the primary evidence … [but] what is required is genuine and meaningful disclosure of the reasons why [the decision was made].^68^

General statements about the prisoner’s behaviour or risk were not therefore held to be sufficient. It thus seems highly likely that it would not be permissible for a public authority to use an ADM system which operated in too ‘black box’ a fashion in a circumstance where the law would usually require ‘gisting’ (such as where, for example, the claimant’s liberty was at stake).^69^

This is borne out by the decision in *Houston Federation of Teachers v Houston Independent School District*^70^ (HISD), which concerned the use by HISD of Educational Value-Added Assessment System (EVAAS) scores generated by a privately developed algorithm. The teachers argued that they had no meaningful way to ensure that their scores had been calculated correctly, even though these scores could be used to terminate their contracts for ineffective performance. In response to this claim, HISD asked for summary judgment against the teachers, but this was denied on the basis that ‘HISD teachers have no meaningful way to ensure correct calculation of their EVAAS scores, and as a result are unfairly subject to mistaken deprivation of constitutionally protected property interests in their jobs’.^71^ Once the claimants had thus been permitted to proceed to trial, the case settled and HISD stopped using the EVAAS scores.^72^

In France, legislation has gone even further, requiring users of ADM to release their source code, in addition to which the Digital Republic Law requires the publication of any source code used by the government. In a rules-based system this is likely to be very helpful, while in a machine-learning context it may well be much less so.^73^

In sum, it seems likely that, in the future, actions will be brought to challenge both the level of transparency of a particular ADM system and the closely connected question of the choice of that particular system rather than another one which may have been more transparent. The courts will therefore have to continue the work they have begun in the context of CMPs,^74^ among other things to establish precisely how much transparency is necessary in different contexts, and when, if ever, as the court in *Houston* put it, ‘cost considerations’ should be allowed to ‘trump accuracy’ (or, one might add, any of the other metrics detailed above).

### B. Jurisdiction

As is well known, the rules relating to jurisdiction require that the public authority should interpret correctly any conditions precedent to the exercise of its power. Thus, if the statute provides that ‘if [X event occurs] the public authority may or shall do [Y thing]’, the public authority must begin by assessing correctly whether or not X event has actually taken place. But does that still hold if the presence of such a precedent X condition is determined by ADM rather than by a human decision maker?

In part, the answer depends on the nature of the system. While ML ADM may operate as a ‘black box’,^75^ rules-based systems do not,^76^ and in the case of a rules-based system it will first be necessary to give the system a set of criteria to use in deciding whether or not the X condition has been fulfilled. This process will still require human judgment, which is likely to continue to attract deference in an orthodox way. Perhaps counterintuitively, however, although we are used to the possibility of ADM rendering decision making less transparent, the process of having to specify these criteria to a machine through a rules-based system might actually make it more transparent and more conscious. This process of specifying the relevant criteria to a rules-based system may therefore mean that there is potentially more material for a court to review than there would be for a human decision maker. The same may also apply to the transparency of the input variables in a regression model. In fact, even when an ML system is used, if it is a system based on ‘supervised learning’^77^ it will be given a set of labelled data, and these labelling choices may also be more transparent and thus open to review.

The overall result, therefore, may well be a reduction of deference, as what is currently decided broadly, implicitly and even unconsciously by humans within a general sphere of discretion becomes instead a series of detailed, explicit technical decisions which are, by the very fact of their transparency, more open to judicial review. Even so, this seems an inevitable part of the process of automation and a necessary price to pay.

However, if the ADM does use ML, an additional set of issues arises.

The decision in *South Yorkshire Transport*^78^ suggests that one way of ensuring that the appeal/review distinction is maintained in judicial review of jurisdiction is for the court to specify the definition of an X condition to within a reasonable range and then leave it for the public authority to make its own decision within that range. But which of the different metrics listed above should be prioritised in assessing the system’s performance in order to decide whether it was ‘reasonable’ in *South Yorkshire*? Will it be a determination by a system that performs well on sensitivity? On accuracy? On precision? What factors should lead to the choice of one of these metrics rather than another? And these are only some of the potentially relevant metrics.^79^ We have already seen that in *Bridges*^80^ the Court of Appeal held that the assessments of accuracy and in particular bias (which might be thought of as uneven accuracy across subgroups) conducted by SWP were inadequate under the PSED. It seems likely to be only a matter of time before there is also a challenge to the metrics used to assess whether an ADM system has made a correct decision about the fulfilment or otherwise of an X condition in a statute for the purposes of establishing the jurisdiction of the public authority.

These are novel questions which until now the law has not had to address, but it seems inevitable that if public authorities continue to make use of ADM, such issues will arise for judicial review sooner or later. And when they do, as *Bridges* demonstrated, it will be vital for courts to have a detailed grasp of the technical matters as well as the legal doctrine, in order to answer them in a manner which provides helpful guidance for those operating the systems as well as necessary protection for those subject to them.

There is, naturally, no inherent reason why ML ADM should be less accurate than the human it augments or replaces; quite the contrary.^81^ However, there are two reasons why we should be more wary of any errors such a system might make.

The first arises from the way in which such systems are created, as identified in *Bridges*. It is obviously highly likely that human decision makers will have implicit biases which lead to the creation of vicious cycles. But crucially, this is unintentional. Human decision makers are in principle trained and intended to make objective judgments, to which their biases are an impediment that arises accidentally. The difficulty with ADM systems, however, is that they are trained using existing data, with the result that any bias or societal inequality which currently exists in the world as a result of those biased human decisions is explicitly baked into their creation. It is the bias in these data, as opposed to a series of objective principles, which they are specifically taught to replicate. This still does not necessarily mean that they will end up being ‘more’ biased than human decision makers, but it does mean (i) that our certainty that biased outcomes will happen is even greater than it is in relation to human decision makers; and (ii) that, while there can and should, of course, be duties placed on human decision makers to undergo training to try to rid them of their biases, unconscious or conscious, we know much less about the extent of such human bias, or the likely success of methods that might be used to eliminate it. For an ADM system, on the other hand, we can measure this bias accurately and measure directly the success or otherwise of our attempts to avoid it.^82^ And the fact that we can do these things increases the duty on us to do so.

Secondly, there is also the concern that if such a system does have a flaw, its impact will be widely felt. As the AI Now Institute at New York University has explained, a flawed ADM system may be used on a much greater scale than the decisions of any individual human. Even if the ADM system generates a lower percentage of errors (wrong decisions), it will make many more decisions overall and thus more people will be affected.^83^ And, if anything, the fact that there may even be fewer (but more significant) errors in an ADM system means that the onus to identify and correct them is greater.

It is in addition the case, particularly though not exclusively with unsupervised learning, that an ML system will determine, on the basis of its own criteria, when a particular X condition in a statute has been fulfilled. This will also raise the question of the factors it took into account in making that determination, a matter which is relevant to another ground of review dealing with precisely that issue.

### C. Taking the Right Things into Account

As is evident from some of the quotes in the section on reason giving above, one key reason why transparency of reasons is vital is because it is then possible to challenge the factors that were taken into account by a decision maker in reaching the decision.^84^ This will ensure that decision makers have not acted for improper purposes,^85^ or on the basis of irrelevant considerations,^86^ while ensuring that they have taken into account anything relevant. The weight to be given to the consideration is thereafter for the decision maker to choose.^87^ It is obvious that this ground of review has the potential to be very relevant to review of ADM, especially in the context of potential ‘bias’, or more accurately discrimination in such systems. This is especially so given that *Wednesbury* itself gave the example of reliance on an impermissible, discriminatory characteristic (red hair) as something that it would prohibit.^88^

There are two potential stages at which this issue of relevance is likely to arise, first where a human decision maker has to establish how much relevance to accord to the result of a determination by an ADM system, and second because the ADM system itself will have taken various factors into account in producing a determination, either to give to a human or to execute on its own. This demonstrates that the law needs a successful, clear and certain account of what is relevant and what is not in different circumstances. We are, however, a long way from this. Thus, for example, it is sometimes possible to take financial cost into account as a relevant factor in making a decision,^89^ while at other times it is not,^90^ but it is not immediately obvious when or why. If the law is thus currently unable to be clear on a relatively familiar factor such as economic cost, it seems that it will face even more challenges as it deals with ADM.

If, for example, a human decision maker takes the output of an ADM system into account, then whether or not that output was indeed relevant may well turn on its quality, particularly in the case of ML systems. But as detailed above, that quality can be measured by a variety of different metrics, not all of which may point in the same direction. Here, then, we face the same challenge as before: is an ADM output to be considered relevant when it is accurate? Precise? Sensitive? Or some other metric? Again, it seems likely that different metrics will be appropriate and will render the system more or less appropriate in different contexts.^91^ There is thus work for public law to do in establishing which of these metrics should apply in each case.

Conversely, at present, once a factor is regarded as being relevant, the precise weight to be given to that factor is for the decision maker to choose.^92^ However, in the context of the PSED, we can already see that this is not the case for some considerations relating to equality of certain protected categories. And it is not impossible to imagine that we may want to extend beyond this in the common law. If, for example, it is apparent that an ADM system is having a particularly problematic effect in relation to a certain category of people, then, even if they are not protected by the PSED, we might well ask whether this is a relevant factor that we would like the decision maker positively to take into account in assessing the performance and thus relevance of the ADM system. And we might want to do this with less deference to decision makers than they receive under the *Tesco*^93^ doctrine.

As for the ADM system itself, everything depends, again, on the precise kind of system. As was noted above in relation to the ground of jurisdiction,^94^ for some kinds of system in some ways these factors might actually be rendered more transparent in the context of ADM than they are for human decision making, since they will be the result of conscious choices in training the system, labelling the data used for regression, establishing the rules for a rules-based system, etc.

However, where ML is used, it relies on statistical inferences, not reasoning, so when such a system uses a particular factor it is not identifying that factor as relevant to the decision it is making in the way that a human would, but merely recognising that, statistically, that factor often correlates with the relevant outcome. For example, a marketing company was able to use YouGov profile data to establish the top 10 brands favoured by ‘Brexiters’ and ‘Remainers’ in the 2016 Brexit Referendum.^95^ It is difficult to imagine that the side one took in this referendum could possibly be a relevant consideration for a public law decision, but let us assume for a moment that it could. If an ADM system were to be created to make this hypothetical decision and it were to make the same calculation that HP Sauce was the number one brand for Brexiters and thus purchase of this brand was highly correlated with Brexit voting, would it then be permissible for that ADM system to use HP Sauce purchase as a relevant consideration in and of itself? From a human perspective, where we hope that the relevance of our considerations is based on their causal contribution to the desired outcome, such an approach would seem impermissible, yet if we were to focus only on the accuracy of the system, an ADM system might well outperform a human decision maker as it can do in other contexts. It is clear, then, that in navigating these kinds of issues the law of (ir)relevance will need to be considerably more sophisticated than it is at present.

A second point is that, regardless of the precise type of system in use, it will be important to establish that the right individuals are responsible for making the relevant choices. On the one hand, choices made in designing the system might be seen as purely technical, and appropriate for those designing and producing the system, as long as the output of the system is successful in relation to the relevant metrics as outlined above. Those making these ‘technical’ decisions will in all likelihood be, as in the case of COMPAS, EVAAC and AFR Locate, private companies. But conversely, as the decision in *Bridges*^96^ demonstrates, the duties of public authorities extend not just to the outputs of such systems, but also to their inputs. It is therefore vital that genuine policy decisions about relevant factors are made by public authorities and that those authorities are held accountable for those choices in the usual way. If this means that public authorities need to go further ‘upstream’ in their investigation, or even design of the systems prior to purchase, as in *Bridges*, and if this means that their decisions on this front are the subject of slightly less deference by the courts, this seems a necessary price to pay, given the possible loss of accountability that might otherwise result. This, in turn, leads us to consider a further relevant ground of review.

### D. Fettering and Non-delegation

We know that a public authority cannot re-delegate power that has been delegated to it, while at the same time the many and varied burdens on public authorities, and their senior officials in particular, mean that total avoidance of delegation is unrealistic.^97^ Similarly, courts have recognised the benefit of policies in terms of consistency and efficiency, and so have sought to strike a balance, as in *Ivory Coast*,^98^ in holding that public authorities must not ‘fetter their discretion’, binding themselves too stringently by either contract^99^ or inappropriately rigid policies.^100^ This has led to a spectrum of responses by the court, so that although the orthodoxy is the *British Oxygen*^101^ position that decision makers must not ‘close their ears’ to other considerations, in some cases the courts have required the policy to play an even smaller role than this,^102^ while in others, even in a purely human decision-making context, courts have permitted a fairly automatic application of the policy.^103^

These grounds have a number of implications for public authority use of ADM, regardless of the particular technology at issue. First, as noted above, it will be important to ensure that even if the ADM system is supplied by a private company, this does not remove or displace the accountability of the public authority, as indeed was made clear by the Court of Appeal in *Bridges*.^104^ Secondly, these grounds suggest that it will be difficult for decision makers to justify relying exclusively on algorithms, in the sense that the human decision maker would essentially follow the suggested outcome without further consideration.^105^ Exclusive reliance could, of course, be specified as required in primary legislation, such as the legislation required by section 49 of the DPA 2018 or section 22(b) of the GDPR. However, such legislation would need to contain ‘suitable measures to safeguard the data subject’s rights, freedoms and legitimate interests’. This might, in turn, suggest the use of the doctrines against fettering and delegation.

Even where there is such human involvement, this may well not be enough. Far from seeing the presence of a ‘human failsafe’ as a minimum requirement in *Bridges*,^106^ the Court of Appeal was unpersuaded that their presence made any difference at all. Noting that the Divisional Court had been particularly impressed by the fact that no intervention would take place without two human beings, including at least one police officer, deciding to act on a positive AFR match, the Court of Appeal gave three reasons for its very different view: first, because the PSED is about the process that needs to be followed, not whether or not the right outcome was reached in a particular case; second, because ‘human beings can also make mistakes. This is particularly acknowledged in the context of identification’; and third, because in any event the relevant matter was that ‘SWP had not obtained information for themselves about the possible bias which the software they use may have’. In other words, SWP’s PSED in this particular case was, as outlined above, to ascertain whether or not the training data for the AFR system had been biased. This process-based duty could not be discharged or avoided by ensuring that the *outcome* of such a system was checked by a human, not least because that human might themselves also be mistaken.

This potential for mistakes leads us to the second implication of the non-delegation, non-fettering doctrines, which is that even where there is human involvement the person concerned should be careful not to over-rely on the technology. Automation bias and automation complacency (respectively a tendency of non-computer scientists to give greater credence to, and thus over-rely on information supplied by technology even when other sources of information differ; and a tendency to monitor technology with less vigilance because of a lower suspicion of error and stronger belief in its accuracy) have been well documented in other fields^107^ and the non-delegation, non-fettering doctrines are useful tools to enable public law to guard against them.

This fits with the decision of the Supreme Court of Wisconsin in *Loomis*,^108^ which concerned a defendant who had been sentenced *inter alia* on the basis of a risk score generated by COMPAS. COMPAS is a tool designed to assess offenders’ criminogenic status and risk of recidivism. It was created by a private firm, Northpointe,^109^ and is used by a number of law enforcement agencies across the United States. The Supreme Court of Wisconsin held that that ‘risk scores [generated by COMPAS] may not be used as the determinative factor in deciding whether an offender can be supervised safely and effectively in the community’,^110^ nor to determine whether an offender should be incarcerated or to determine the severity of the sentence. Conversely, it held that ‘a circuit court must explain the factors in addition to a COMPAS risk assessment that independently support the sentence imposed. A COMPAS risk assessment is only one of many factors that may be considered and weighed at sentencing.’^111^

Similarly, in England, the fact that the Resource Allocation Systems^112^ used by local councils for allocating disability benefits were ‘only a starting point’ and ‘sufficiently flexible’ was a key factor in establishing their legality.^113^

Indeed, there is a danger more generally that use of ADM may encourage de-individualisation and rigidity of policies.^114^ This may at first seem counterintuitive; after all, one of the key aims of ‘big data’ is to develop *more*, not less, precise or tailored solutions. Thus, for example, we are familiar with the idea of personalised or micro-targeted marketing^115^ and precision medicine.^116^ There has even been a suggestion that the law should move in a similar direction to personalise rules such as the standard of negligence.^117^ However, there are some fundamental differences between these examples. In the case of medicine, as noted above, while data might be used to identify a potential therapy, it is much less likely that that therapy will be applied to a particular patient without an additional check to ensure that the patient or their disease does indeed have the relevant genetic code. Thus, for example, research has established that vemurafenib works only in the treatment of patients whose cancer tests positive for the V600E BRAF mutation. But individual patients are tested to see whether or not they do indeed have this mutation before being offered a course of the drug.^118^ By contrast, in the case of targeted marketing, or even proposed tailored legal regimes, there is no such individualised case-specific stage of testing; once a system predicts that a person will fulfil a particular criterion, there is no further test, and it may well not even be possible to test whether that person does indeed belong in that category. Thus, while on one level ‘tailored’ regimes sound seductively individual, outside medicine they are individual only up to a point. It is therefore entirely possible that an *automated* decision-making system in the executive context,^119^ which is individualised at a macro level but only to this limited extent, may still be less individual at a micro level than a decision by a human decision maker who has a default general rule that they may depart from if the particular case requires it. In this context, it is interesting that in the case of the A level results an automated system based on aggregate data was ultimately abandoned in favour of individualised teacher assessments.^120^ A further distinction between the use of micro-targeting in medicine (and micro-targeted adverts) and the use of data in public authority executive decision making is the ability of the patient or claimant to consent. Thus, even in a context in which the science does still have to operate at a more macro level, because we do not yet know who may or may not benefit from a treatment, it is still very much the patient’s own choice whether to take the small chance of a highly successful outcome set against the greater chance of a fairly toxic and potentially ineffective treatment.^121^ In contrast, as was noted at the outset, consent cannot really operate in a public authority executive decision making case.^122^

It follows that, unless the micro-targeting is as accurate as case-by-case individual decision making, it remains distinct from true individualisation, and it is that distinction which must be preserved by the doctrines against fettering. If it is not preserved, then the risk is that once an ADM system is in place it will inevitably pull decision makers towards the *Ivory Coast* end of the review spectrum, in which the efficiency and speed of the routine and automated system are prioritised, even if they lead to injustice or unfairness in individual cases.^123^ And if we combine this with the points made above, those most likely to suffer such injustices are also those most likely to be already at a disadvantage. This is precisely because it is those existing disadvantages which are baked into the system through the use of data.

### E. Substantive Checks: Reasonableness and Proportionality

It is also possible that ADM will be challenged using the overlapping checks of *Wednesbury*^124^—unreasonableness and proportionality.^125^ We have already seen that proportionality is specifically mandated in certain contexts, such as the handling of article 9 GDPR (sensitive) data. These grounds may again be relevant at two different stages in relation to ADM: first, the decision to use a particular ADM system; and second, reviewing the output of such a system.

It is well known that the ‘necessity’ aspect of proportionality essentially asks whether a sledgehammer has been used to crack a nut. A key question in choosing ADM in the first place, therefore, will be whether the ADM is a sledgehammer and whether, for example, augmented decision making might not have been better than full ADM. There may also be a ‘fair balance’ issue, going to the final aspect of proportionality. This would require an examination of the benefits of the ADM when set against its impacts and potential disadvantages, such as the potential lack of transparency or its potentially detrimental effect on minority classes of people (even outside the PSED). The ICO’s guidance seems likely to be particularly relevant here, stating that:

when planning on using AI to help make decisions about people, you should consider:

1. The setting in which you will do this;
2. The potential impact of the decisions you make;
3. What an individual should know about a decision, so you can choose an appropriately explainable AI model; and
4. Prioritising delivery of the relevant explanation types.^126^

In terms of reviewing the output of an ADM system, suitability in the proportionality context is relatively weak; as long as there is ‘not nothing’ to connect the means sought to the ends used, the measure may survive challenge.^127^ However, counter-intuitively, in the rationality context suitability can in fact be stronger, so that in *Wandsworth*^128^ the court struck down a measure for being ‘not rationally capable’ of achieving the relevant aim and because the connection between means and ends was ‘highly speculative’. Similarly, in the *Law Society*^129^ case, the measure failed because it was found to do more harm than good. It does therefore seem that such grounds might, for example, provide a helpful way to challenge pernicious feedback loops^130^ where the system in fact creates the very thing it is supposed to be detecting. One example would be where a system predicts recidivism, leading to imprisonment rather than a community sentence, when it is well known that in fact incarceration increases the chances of recidivism. Such loops are, after all ‘not rationally capable of helping’, and do ‘make things worse not better’. It is likely that these less deferential approaches may be necessary if public law is to be able to exercise the necessary control here.

It is also worth noting that if an ADM is discriminatory it will also in all probability not be necessary for proportionality purposes. If it is not necessary to treat one group in such a disadvantageous way, how can it be necessary for another group?^131^

Here too, then, it appears that not only is it necessary to clarify the operation of substantive review for current purposes,^132^ it is even more necessary if the ground is to be sufficiently clear and precise to apply in order to guide the optimal use of ADM.

## 9. Conclusion

There is no question that the ability to base public decision making on data rather than personal judgment has great potential in terms of accuracy and objectivity. And the ability to automate this process can render decision making more consistent, predictable and efficient. However, as outlined here, a move to ADM also raises a number of challenges in the form of technical decisions about the necessary metrics for evaluating such systems, opacity, scalability of errors and so on. If it is to provide the necessary guidance to enable optimal use of such systems, there are a number of ways in which administrative law needs to become more nuanced and advanced. If it is able to do this, administrative law has the potential not only to do useful work itself in controlling ADM, but also to support the work of the ICO and provide guidance on the interpretation of concepts such as ‘meaningful information’ and ‘proportionality’ within the GDPR. And given that one of the key determinants of the application of public law is the imbalance of power between the decision maker and the private individual,^133^ taken alongside the potential for ‘leakage’ of public law outside its traditional boundaries,^134^ there may even be a role for public law in the control of ADM more widely.

## Footnotes

1 B Staton and L Hughes, ‘A-level and GCSE students to have downgraded results restored’ *Financial Times* (London, 17 August 2020) <www.ft.com/content/273ff590-9651-4e25-aaa4-157d5b2948e1> accessed 17 May 2021.

2 See also L Dencik, A Hintz, J Redden and H Warne, ‘Data Scores as Governance: Investigating Uses of Citizen Scoring in Public Services. Project Report’ (Data Justice Lab, Cardiff University, December 2018) <https://datajustice.files.wordpress.com/2018/12/data-scores-as-governance-project-report2.pdf> accessed 17 May 2021; M Oswald, ‘Algorithm-Assisted Decision-Making in the Public Sector: Framing the Issues Using Administrative Law Rules Governing Discretionary Power’ (2018) 376 Philosophical Transactions of the Royal Society 2128, especially fn 7. See also J Maxwell and J Tomlinson, ‘Public Law and Technology: Mapping and Analysing Legal Responses in UK Civil Society’ [2020] PL 28, [24]. For a US perspective, see D Freeman Engstrom and others, ‘Government by Algorithm: Artificial Intelligence in Federal Administrative Agencies’ (report submitted to the Administrative Conference of the United States, February 2020) <https://law.stanford.edu/education/only-at-sls/law-policy-lab/practicums-2018-2019/administering-by-algorithm-artificial-intelligence-in-the-regulatory-state/acus-report-for-administering-by-algorithm-artificial-intelligence-in-the-regulatory-state/#slsnav-report> accessed 17 May 2021. An investigation in *The Guardian* in 2019 found them in use in 140 of 408 councils in the UK: S Marsh ‘One in Three Councils Using Algorithms to Make Welfare Decisions’ *The Guardian* (London, 15 October 2019) <www.theguardian.com/society/2019/oct/15/councils-using-algorithms-make-welfare-decisions-benefits> accessed 17 May 2021.

3 C O’Neill, *Weapons of Math Destruction* (Penguin Random House USA 2016).

4 F Pasquale, *The Black Box Society: The Secret Algorithms that Control Money and Information* (Harvard UP 2015).

5 S Marsh, ‘Councils Scrapping Use of Algorithms in Benefit and Welfare Decisions’ *The Guardian* (London, 24 August 2020) <www.theguardian.com/society/2020/aug/24/councils-scrapping-algorithms-benefit-welfare-decisions-concerns-bias> accessed 17 May 2021.

6 J Naughton, ‘From Viral Conspiracies to Exam Fiascos, Algorithms Come with Serious Side-Effects’ *The Guardian* (London, 6 September 2020) <www.theguardian.com/technology/2020/sep/06/from-viral-conspiracies-to-exam-fiascos-algorithms-come-with-serious-side-effects> accessed 17 May 2021. See also *Patullo v HMRC* [2016] UKUT 270, [2016] BTC 510; *R (Barking and Dagenham College) v The Office for Students* [2019] EWHC 2667, [2020] ELR 152; *R (W) v Birmingham* [2011] EWHC 1147, [2011] Eq LR 721.

7 A Hern, ‘Ofqual’s A-Level Algorithm: Why Did It Fail to Make the Grade?’ *The Guardian* (London, 21 August 2020) <www.theguardian.com/education/2020/aug/21/ofqual-exams-algorithm-why-did-it-fail-make-grade-a-levels> accessed 17 May 2021.

8 See eg Kadioglu O, Saeed M, Johannes Greten H & Efferth T. Identification of novel compoundsagainst three targets of SARS CoV-2 coronavirus by combined virtual screening and supervisedmachine learning. [Preprint]. Bull World Health Organ. E-pub: 21 March 2020. doi:http://dx.doi.org/10.2471/BLT.20.25594; S Whitelaw and others, ‘Applications of Digital Technology in COVID-19 Pandemic Planning and Response’ (2020) 2 The Lancet Digital Health 435.

9 On which see further T Melham and R Williams, *Practical Law Public Sector* 8 December 2020.

10 RK Hill, ‘What an Algorithm Is’ (2015) 29 Philosophy & Technology 35.

11 S Bhatti and others, ’Algorithms in the Criminal Justice System: A Report by the Law Society Commission on the Use of Algorithms in the Justice System’ (The Law Society of England and Wales, June 2019) 10.

12
*Sharman v HM Coroner for Inner North London* [2005] EWCA Civ 967, [2005] Inquest LR 168. See also V Mayer-Schönberger and K Cukier, *Big Data* (John Murray 2013) 73–6. See also M Veale and I Brass, ‘Administration by Algorithm? Public Management Meets Public Sector Machine Learning’ in K Yeung and M Lodge (eds), *Algorithmic Regulation* (OUP 2019); C Coglianese and D Lehr, ‘Regulating by Robot: Administrative Decision Making in the Machine-Learning Era’ (2017) 105 Geo LJ 1147.

13 See eg the ICA1 form algorithm for prisoner categorisation referred to in *R (Guittard) v Secretary of State for Justice* [2009] EWHC 2951; and PSI-17-2006, referred to in *R (Lewis) v HM Coroner* [2009] EWHC 661, (2009) 108 BMLR 87.

14 See eg J Schmidhuber, ‘Deep Learning in Neural Networks: An Overview’ (2016) 61 Science Direct 85.

15 For an outline of different algorithmic techniques with different levels of transparency, see The ICO and the Alan Turing Institute, ’Explaining Decisions Made with AI’ (20 May 2020) Annexe 2 <https://ico.org.uk/for-organisations/guide-to-data-protection/key-data-protection-themes/explaining-decisions-made-with-ai/> accessed 17 May 2021.

16 Also sometimes called ‘recall’.

17 S M McKinney and others, ’International evaluation of an AI system for breast cancer screening’ (2020) 577 Nature 89.

18 Above n 1, 13.

19 ibid.

20 Above n 3, 98.

21 Above n 3, 27.

22 R Brauneis and E Goodman, ’Algorithmic Transparency for the Smart City’ (2018) 20 Yale Journal of Law & Technology, 103, 112.

23 O Eren and N Mocan, ‘Emotional Judges and Unlucky Juveniles’ (2018) 10(3) American Economic Journal: Applied Economics 171.

24 See the doctrine of legitimate expectations in administrative law, especially as discussed in the case of Mandalia v Secretary of State for the Home Department [2015] UKSC 59, [2015] 1 WLR 4546.

25 See further the evidence of Kriti Sharma, quoted by the House of Lords Select Committee on Artificial Intelligence, *AI in the UK: Ready, Willing and Able?* (HL 2017-19, 100-I) [118]<https://publications.parliament.uk/pa/ld201719/ldselect/ldai/100/100.pdf> accessed 17 May 2021. See also J Cobbe, ‘Administrative Law and the Machines of Government: Judicial Review of Automated Public-Sector Decision-Making’ (2019) 39 LS 636.

26 Regulation (EU) 2016/679 of the European Parliament and Council [2016] OJ L119/1 (GDPR).

27 Case 215/88 *Casa Fleischhandels* [1989] ECR 2789 [31].

28 The Keeling Schedule for which can be found at <www.gov.uk/government/publications/data-protection-law-eu-exit> accessed 17 May 2021.

29 Though amendments are pending which will make the section refer to art 22 of the GDPR instead.

30 Article 29 EC Data Protection Working Party, ‘Guidelines on Automated Individual Decision-Making and Profiling for the Purposes of Regulation 2016/679’ COM (2018) 26.

31 B Goodman and S Flaxman, ‘EU regulations on Algorithmic Decision-Making and a “Right to Explanation”’ (2017) 38(3) AI Magazine 50.

32 S Wachter, B Mittelstadt and L Floridi, ‘Why a Right to Explanation of Automated Decision-Making Does Not Exist in the General Data Protection Regulation’ (2017) 7 IDPL 76, 83; and see also 84.

33 A Selbst and J Powles, ‘Meaningful Information and the Right to Explanation’ (2017) 7 IDPL 233.

34 M Kaminski, ‘The Right to Explanation, Explained’ (2019) 34 Berkeley Technology Law Journal 189.

35 Above n 30, 25–6.

36 Above n 14.

37 ibid 20, *inter alia*.

38 ibid 21.

39 Above n 30, 17.

40 ibid 27.

41
*R (Bridges) v Chief Constable of South Wales Police* [2020] EWCA Civ 1058, [2020] 1 WLR 5037.

42 Actions had previously been threatened in response to the A level decisions <www.foxglove.org.uk/2020/08/17/we-put-a-stop-to-the-a-level-grading-algorithm/> accessed 14 September 2021) and the visa streaming tool (/www.foxglove.org.uk/2020/08/04/home-office-says-it-will-abandon-its-racist-visa-algorithm-after-we-sued-them/> accessed 14 September 2021; H McDonald, ‘AI System for Granting UK Visas Is Biased, Rights Groups Claim’ *The Guardian* (London, 29 October 2019) <www.theguardian.com/uk-news/2019/oct/29/ai-system-for-granting-uk-visas-is-biased-rights-groups-claim> accessed 18 May 2021). Neither case was heard because the systems had in the meantime been abandoned.

43 See also the Dutch case ECLI:NL:RBDHA:2020:1878, a decision by the Hague District Court that a system called SyRI used by the Dutch government to detect various forms of fraud, including social benefits, allowances and tax fraud, was in breach of art 8 ECHR.

44 For definitions of these terms, see further above n 16 and surrounding text.

45 Above n 41, [199].

46 ibid.

47 ibid [193].

48 See further R Binns, ‘Algorithmic Decision-Making: A Guide for Lawyers’ (2020) 25 JR 2.

49
*R v Secretary of State for the Environment ex p Hammersmith and Fulham LBC* [1991] 1 AC 521, 561 (Lord Donaldson).

50 See eg *Moyna v Secretary of State for Work and Pensions* [2003] UKHL 44, [2003] 1 WLR 1929; *Pendragon Plc v Revenue and Customs Commissioners* [2015] UKSC 37, [2015] 1 WLR 2838, [49]–[51]; *R v Monopolies and Mergers Commission ex p South Yorkshire Transport* [1993] 1 WLR 23 (HL).

51 See eg *Carltona v Commissioners of Works* [1943] 2 All ER 560; *R (CCWMP) v Birmingham Justices* [2002] EWHC 1087, [2002] Crim LR 37; *R v Inhabitants of Leake* (1833) 5 B & Ad 469; *British Oxygen v Minister of Technology* [1971] AC 610.

52 See eg *Dimes v Grand Junction Canal Co* (1852) 3 HLC 759; *R v Amber Valley DC, ex p Jackson* [1985] 1 WLR 298; *Ex p Kirkstall Valley* [1996] 3 All ER 304; *R v UFC, ex p Institute of Dental Surgery* [1994] 1 WLR 242; M Elliott, ‘Has the Common Law Duty to Give Reasons Come of Age Yet?‘ [2011] PL 56; *Secretary of State for the Home Department v AF (No 3)* [2009] UKHL 28, [2010] 2 AC 269, following *A v United Kingdom* (2009) 49 EHRR 625 (GC) and *R (Bourgass) v Secretary of State for Justice* [2015] UKSC 54, [2016] AC 384.

53
*R v Governor of Brixton Prison ex p Soblen* [1963] 2 QB 243, 302; *Bancoult* [2013] EWHC 1502, [2014] Env LR 2; *R v Rochdale MBC ex p Cromer Ring Mill* [1982] 3 All RR 761; *R v Westminster Corporation v LNWR* [1905] AC 426; *Tesco Stores v Secretary of State for the Environment* [1995] 1 WLR 759.

54
*Associated Provincial Picture Houses v Wednesbury Corporation* [1948] 1 KB 223 (CA); *Ex p Barry* [1997] AC 584; *Tandy* [1998] AC 714; *R (Quila) v Home Sec* [2011] UKSC 45, [2012] 1 AC 621; *R (Wandsworth v Schools Adjudicator*) [2003] EWHC 2969, [2004] ELR 274; *R (Law Society v Legal Services Commission*) [2010] EWHC 2550, [2011] ACD 16; *A v Home Secretary* [2004] UKHL 56, [2005] 2 AC 68.

55 Above n 15, 57.

56 GDPR, art 9(2)(g) and (j). See also eg art 14(5)(b), art 19.

57 The initial right of recourse against the ICO is to the First-tier Tribunal (General Regulatory Chamber), but DPA 2018, s 163(3) suggests that the grounds of judicial review will be relevant as potential grounds for allowing an appeal. Appeal then goes up through the Upper Tribunal, ultimately, though rarely, leading to judicial review. See eg Information Commissioner’s Office, ‘ICO Disclosure Log—Response IRQ0686975’ (2017) <https://ico.org.uk/media/about-the-ico/disclosure-log/2172601/irq0686975-response.pdf> accessed 11 July 2021.

58
*Dimes* (n 52).

59 See eg the specification that a tribunal must be independent and impartial, under art 6, rather than simply justifiable in its own right as a political decision-making method: *Amber Valley* (n 52); *Kirkstall Valley* (n 52).

60 Above n 14.

61 ibid 48.

62
*EK (Ivory Coast)* [2014] EWCA Civ 1517, [2015] Imm AR 367.

63 See eg *UFC* (n 52); Elliott (n 52).

64 See eg *AF (No 3)* (n 52), following *A* (n 52).

65 Above n 14, 23.

66 ibid.

67
*Bourgass* (n 52).

68 ibid [100].

69 See also Oswald (n 2) text to n 7. It is true that in the US case *State of Wisconsin v Loomis* 2016 WI 68, 371 Wis 2d 235, 881 NW 2d 749 this ground was less successful. But this was not a necessary ground of decision in *Loomis* since the court was in any event finding for the claimant on other grounds (see further below, text to n 108).

70
*Houston Federation of Teachers v Houston Independent School District* 251 F Supp 3d 1168 (SD Tex 2017).

71 ibid.

72 M Cummings, ‘Federal Lawsuit Settled between Houston’s Teacher Union and HISD’ *Houston Public Media* (Houston, 10 October 2017) <www.houstonpublicmedia.org/articles/news/2017/10/10/241724/federal-lawsuit-settled-between-houstons-teacher-union-and-hisd/> accessed 19 May 2021.

73 On the limits of transparency, see further L Edwards and M Veale, ‘Slave to the Algorithm? Why a “Right to an Explanation” Is Probably Not the Remedy You Are Looking For’ (2016) Duke Law & Technology Review 18.

74 See eg the contrast between *AF (No 3)* (n 52) and *Home Office v Tariq* [2011] UKSC 35, [2012] 1 AC 452.

75 Above n 4.

76 See above n 15.

77 As opposed to unsupervised learning, where the system is given unlabelled data which it identifies by extracting features and patterns on its own.

78 Above n 50.

79 See also F-score: the harmonic mean of precision and recall.

80 Above n 41.

81 See above, eg n 23.

82 See eg N Mehrabi and others, ‘A Survey on Bias and Fairness in Machine Learning’ ArXiv Research Paper (arXiv:1908.09635v2 [cs.LG] 2019) <https://arxiv.org/abs/1908.09635> accessed 19 May 2021; D Pedreshi, S Ruggieri and F Turini, ‘Discrimination-Aware Data Mining’ (Proceedings of the 14th Association for Computer Machinery SIGKDD International Conference on Knowledge Discovery and Data Mining, August 2008); C Dwork and others, ‘Fairness through Awareness’ (Proceedings of the 3rd Innovations in Theoretical Computer Science Conference, January 2012); M Hardt, E Price and N Srebro, ‘Equality of Opportunity in Supervised Learning’ (Proceedings of the 30^th^ International Conference on Neural Information Processing Systems, December 2016)

83 M Whittaker and others, ‘AI Now Report 2018’ (AI Now Institute 2018) 18 <https://ainowinstitute.org/AI_Now_2018_Report.pdf> accessed on 19 May 2021.

84 See eg *Governor of Brixton Prison* (n 53) 302; *Bancoult* (n 53).

85
*R v Westminster Corporation v LNWR* (n 53).

86
*Rochdale MBC* (n 53).

87
*Tesco* (n 53).

88
*Associated Provincial Picture Houses* (n 54).

89
*Barry* (n 54).

90
*Tandy* (n 54).

91 Above, text between nn 75 and 78.

92
*Tesco* (n 53).

93 ibid.

94 See above, text to n 75.

95 E James, ‘The Top 10 Brands Favoured by Remainers and Brexiters’ *Campaign* (1 August 2016) <www.campaignlive.co.uk/article/top-10-brands-favoured-remainers-brexiters/1403991> accessed 19 May 2021.

96 Above n 41. See also Oswald (n 2).

97 See eg *Carltona* (n 51); *CCWMP* (n 51).

98 See eg the contrast between Sales LJ and Floyd LJ in *Ivory Coast* (n 62), and note Sales LJ’s explicit attempt to strike a balance at [40]. I am grateful to Jack Beatson for a discussion on this point.

99
*Inhabitants of Leake* (n 51).

100
*British Oxygen* (n 51).

101 ibid.

102
*Stringer* [1970] 1 WLR 1281.

103 See eg *Ivory Coast* (n 62).

104 Above n 41.

105 As appears to have been the case in some of the decided cases, discussed in further detail below, eg *Loomis* (n 69). See also *Houston* (n 70).

106 Above n 41.

107 ISMP Canada, ‘Understanding Human Over-reliance on Technology’ (2016) 16(5) ISMP Canada Safety Bulletin 1; K Goddard, A Roudsari and JC Wyatt, ‘Automation Bias: A Systematic Review of Frequency, Effect Mediators, and Mitigators’ (2012) 19 Journal of the American Medical Informatics Association 121; R Parasuraman and DH Manzey, ‘Complacency and Bias in Human Use of Automation: An Attentional Integration’ (2010) 52 Human Factors 381. See also C Knight, ‘Automated Decision-Making and Judicial Review’ (2020) 25 JR 21; Claimant’s statement of facts and grounds in Foxglove, ‘How We Got the Government to Scrap the Visa Streaming Algorithm—Some Key Legal Documents’ (4 August 2020) pars 53–8 <www.foxglove.org.uk/news/c6tv7i7om2jze5pxs409k3oo3dyel0> accessed 18 May 2021.

108 Above n 69.

109 Northpointe, ‘Practitioners Guide to COMPAS’ (17 August 2012) <www.northpointeinc.com/files/technical_documents/FieldGuide2_081412.pdf> accessed 19 May 2021.

110 Above n 69, paras 44 and 99.

111 This is the same test as was applied to a policy in *Stringer* (n 102).

112 Which at least in some instances involve Quickheart software, see *W* (n 6).

113
*R (Savva) v Royal Borough of Kensington and Chelsea* [2010] EWCA Civ 1209, [2011] PTSR 761; *KM v Cambridge* [2012] UKSC 23, [2012] 3 All ER 1218.

114 See eg Lord Sales, ‘Algorithms, Artificial Intelligence and the Law’ (2020) 25 JR 46, [15].

115 See Q André and others, ‘Consumer Choice and Autonomy in the Age of Artificial Intelligence and Big Data’ (2018) 5 Customer Needs and Solutions 28.

116 See eg M Panahiazar and others, ‘Empowering Personalized Medicine with Big Data and Semantic Web Technology: Promises, Challenges and Use Cases’ (Proceedings of the 14th Association for Computer Machinery SIGKDD International Conference on Knowledge Discovery and Data Mining, August 2008) <www.ncbi.nlm.nih.gov/pmc/articles/PMC4333680/> accessed 19 May 2021; V Gligorijević, N Malod-Dognin and N Pržulj, ‘Integrative Methods for Analyzing Big Data in Precision Medicine’ (2016) 16 Proteomics 741.

117 O Ben-Shahar and A Porat, ‘Personalizing Negligence Law’ (2016) 91 NYU L Rev 628.

118 Cancer Research UK, ‘Vemurafenib (Zelboraf)’ <www.cancerresearchuk.org/about-cancer/cancer-in-general/treatment/cancer-drugs/drugs/vemurafenib> accessed 19 May 2021.

119 ie in non-medical decision-making contexts by officials acting in their capacity as public authority decision makers, rather than by doctors in their professional capacity. See further S Palmer, ‘Public, Private and the Human Rights Act 1998: An Ideological Divide’ (2007) 66 CLJ 559.

120 S Weale and H Stewart, ‘A-Level and GCSE Results in England To Be Based on Teacher Assessments in U-Turn’ *The Guardian* (London, 17 August 2020) <www.theguardian.com/education/2020/aug/17/a-levels-gcse-results-england-based-teacher-assessments-government-u-turn> accessed 19 May 2021.

121 As is the case in some instances of kidney cancer drugs such as Avastin.

122 See above, text to n 27.

123 Above n 62. See also the experience in *Houston* (n 70) in text following n 74.

124 Above n 88.

125 See eg *Bank Mellat v Her Majesty’s Treasury* [2013] UKSC 39, [2014] AC 700.

126 Above n 14, 41.

127
*Quila* (n 54). See also R Williams, ‘Structuring Substantive Review’ [2017] PL 99.

128
*Wandsworth v Schools Adjudicator* (n 54).

129
*Law Society v Legal Services Commission* (n 54).

130 On which see further Melham and Williams (n 9).

131 See eg *A v Home Secretary* [2004] UKHL 56, [2005] 2 AC 68.

132 See further Williams (n 127).

133
*R v Panel on Takeovers and Mergers, ex p Datafin* [1987] QB 815.

134
*Braganza v BP Shipping Ltd and another* [2015] UKSC 17, [2015] 1 WLR 1661.

## Notes

I am very grateful to my colleagues Professor Thomas Melham, Dr Václav Janeček, Professor Paul Craig, Dr Reuben Binns and Dr Jennet Williams for their comments on earlier drafts, as well as to the anonymous reviewers. Any remaining errors are, of course, mine alone.

